# Seroprevalence of herpes simplex virus type 1 and type 2 among the migrant workers in Qatar

**DOI:** 10.1186/s12985-023-02157-1

**Published:** 2023-08-22

**Authors:** Gheyath K. Nasrallah, Soha R. Dargham, Duaa W. Al-Sadeq, Fathima H. Amanullah, Farah M. Shurrab, Parveen B. Nizamuddin, Hiam Chemaitelly, Houssein H. Ayoub, Sami Abdeen, Ashraf Abdelkarim, Faisal Daraan, Ahmed Ismail, Nahid Mostafa, Mohamed Sahl, Jinan Suliman, Elias Tayar, Hasan Ali Kasem, Meynard J. A. Agsalog, Bassam K. Akkarathodiyil, Ayat A. Alkhalaf, Mohamed Morhaf M. H. Alakshar, Abdulsalam Ali A. H. Al-Qahtani, Monther H. A. Al-Shedifat, Anas Ansari, Ahmad Ali Ataalla, Sandeep Chougule, Abhilash K. K. V. Gopinathan, Feroz J. Poolakundan, Sanjay U. Ranbhise, Saed M. A. Saefan, Mohamed M. Thaivalappil, Abubacker S. Thoyalil, Inayath M. Umar, Einas Al Kuwari, Peter Coyle, Andrew Jeremijenko, Anvar Hassan Kaleeckal, Hanan F. Abdul Rahim, Hadi M. Yassine, Asmaa A. Al Thani, Odette Chaghoury, Mohamed Ghaith Al Kuwari, Elmoubasher Farag, Roberto Bertollini, Hamad Eid Al Romaihi, Abdullatif Al Khal, Mohamed H. Al-Thani, Laith J. Abu-Raddad

**Affiliations:** 1 Biomedical Research Center, Qatar University, Doha, Qatar; 2 Department of Biomedical Science, College of Health Sciences, QU Health, Qatar University, 2713 Doha, Qatar; 3 Infectious Disease Epidemiology Group, Weill Cornell Medicine-Qatar, Cornell University, Doha, Qatar; 4 World Health Organization Collaborating Centre for Disease Epidemiology Analytics On HIV/AIDS, Sexually Transmitted Infections, and Viral Hepatitis, Weill Cornell Medicine–Qatar, Cornell University, Qatar Foundation – Education City, P.O. Box 24144, Doha, Qatar; 5 Department of Population Health Sciences, Weill Cornell Medicine, Cornell University, New York, NY USA; 6 Mathematics Program, Department of Mathematics, Statistics, and Physics, College of Arts and Sciences, Qatar University, Doha, Qatar; 7 Hamad Medical Corporation, Doha, Qatar; 8 Ministry of Public Health, Doha, Qatar; 9 Qatar Red Crescent Society, Doha, Qatar; 10 Wellcome-Wolfson Institute for Experimental Medicine, Queens University, Belfast, UK; 11 Department of Public Health, College of Health Sciences, QU Health, Qatar University, Doha, Qatar; 12 Primary Health Care Corporation, Doha, Qatar; 13 College of Health and Life Sciences, Hamad Bin Khalifa University, Doha, Qatar

**Keywords:** HSV-1, HSV-2, Prevalence, Genital herpes, Genital ulcer disease, Survey, Cross sectional study, Migrants, Qatar

## Abstract

**Background:**

Limited data exists on herpes simplex virus type 1 (HSV-1) and type 2 (HSV-2) infections in migrant populations. This study investigated HSV-1 and HSV-2 seroprevalences and associations among craft and manual workers (CMWs) in Qatar who constitute 60% of Qatar’s population.

**Methods:**

A national population-based cross-sectional seroprevalence survey was conducted on the CMW population, all men, between July 26 and September 9, 2020. 2,612 sera were tested for anti-HSV-1 IgG antibodies using HerpeSelect 1 ELISA IgG kits and for anti-HSV-2 IgG antibodies using HerpeSelect 2 ELISA IgG kits (Focus Diagnostics, USA). Univariable and multivariable logistic regression analyses were conducted to identify associations with HSV-1 and HSV-2 infections.

**Results:**

Serological testing identified 2,171 sera as positive, 403 as negative, and 38 as equivocal for HSV-1 antibodies, and 300 sera as positive, 2,250 as negative, and 62 as equivocal for HSV-2 antibodies. HSV-1 and HSV-2 seroprevalences among CMWs were estimated at 84.2% (95% CI 82.8–85.6%) and 11.4% (95% CI 10.1–12.6%), respectively. HSV-1 infection was associated with nationality, educational attainment, and occupation. HSV-2 infection was associated with age, nationality, and educational attainment.

**Conclusions:**

Over 80% of CMWs are infected with HSV-1 and over 10% are infected with HSV-2. The findings highlight the need for sexual health programs to tackle sexually transmitted infections among the CMW population.

**Supplementary Information:**

The online version contains supplementary material available at 10.1186/s12985-023-02157-1.

## Background

Herpes simplex virus type 1 (HSV-1) and type 2 (HSV-2) infections are life-long and common worldwide [1]. Infections with these viruses are typically latent and asymptomatic, but with frequent reactivations, subclinical shedding and sporadic symptomatic events [2–4]. HSV-1 infection in rare cases causes severe neurological, corneal, or mucocutaneous complications [5, 6]. HSV-2 infection causes genital ulcer disease and genital herpes [7, 8], and its vertical mother-to-child transmission can cause neonatal herpes, a severe and sometimes fatal disease in newborns [9, 10]. While HSV-2 is predominantly sexually transmitted [7, 8], HSV-1 is typically orally acquired [11, 12], but with increasing role as a sexually transmitted infection, mainly through oral sex [11, 12]. HSV-2 infection is believed to increase acquisition and transmission of HIV [13], perhaps resulting in a synergy between these two infections [14–16]. The World Health Organization (WHO) and global partners are leading initiatives to enhance our understanding of the epidemiology of these two viruses, and to develop an HSV vaccine to tackle the global disease and economic burden of HSV-1 and HSV-2 infections [1, 17, 18].

Qatar, a country in the Arabian Peninsula, has unusually young, diverse demographics, in that only 9% of its residents are ≥ 50 years of age, and 89% are expatriates from over 150 countries [19]. Nearly 60% of the population comprises expatriate craft and manual workers (CMWs) who are typically single men aged 20–49 years and recruited to work in large development projects, such as those for the World Cup 2022 [20]. The objective of this study was to assess the antibody prevalence (seroprevalence) of HSV-1 and HSV-2 infections among this majority segment of Qatar’s population and to inform national health policy planning.

## Methods

### Study design and sampling

This study was conducted on blood sera specimens that were originally collected for a national severe acute respiratory syndrome coronavirus 2 (SARS-CoV-2) seroprevalence survey among CMWs in Qatar [20]. The blood specimens were drawn between July 26, 2020 and September 09, 2020, to assess exposure to SARS-CoV-2 infection in this population, the most affected population by the first SARS-CoV-2 wave in Qatar [19, 21].

To optimize sample representativeness of the wider CMW population, a sampling strategy was devised based on analysis of the registered users’ database of the Qatar Red Crescent Society (QRCS), the main provider of primary healthcare for CMWs in the country [20]. QRCS operates four geographically distributed centers that were purposively designed to cater to the CMW population across the country. These centers operate long working hours, are located in regions where workers live, and provide services that are free of charge or heavily subsidized for accessibility and affordability.

The probability distribution of CMWs by age and nationality in the QRCS database was cross-checked and found similar to that of the Ministry of Interior database of expatriate residents [22]. Sex was not considered in the sampling strategy because the vast majority of CMWs (> 99%) are men [23]. Systematic sampling was implemented for the recruitment of the CMWs attending these centers during the study [20]. By factoring the average number of attendees per day at each of these centers, every 4th attendee visiting each center was approached to participate in this study until the sample size by age and nationality at each center has been fulfilled. There was difficulty in recruiting participants in the small age-nationality strata (such as among younger persons for specific nationalities), and thus towards the end of the study all attendees in these strata (not only every 4th attendee) were invited to participate. Written informed consent was collected from all study participants.

### Sample collection and handling

An interview schedule including socio-demographic variables was administered by trained interviewers in the participant’s language of preference [20]. The study instrument was based on a protocol for SARS-CoV-2 sero-epidemiological surveys developed by the WHO [24]. Both informed consent and interview schedule were provided and collected in nine languages (Arabic, Bengali, English, Hindi, Nepali, Sinhala, Tagalog, Tamil, and Urdu) to cater to the main language groups of CMWs. Blood (10 ml) was drawn for serological testing by certified nurses and stored in an ice box before being transported to the Qatar Biobank for storage and subsequent testing.

### Laboratory methods

Portion of sera (50 µL) were aliquoted from stored sera samples at the Qatar Biobank and transported to the virology laboratory at Qatar University for the serological testing. The sera at both Qatar Biobank and Qatar University were stored at − 80 °C until used for serology testing. Sera were tested for the presence of anti-HSV-1 IgG using HerpeSelect 1 enzyme linked immunosorbent assay (ELISA) kits (Cat. No. EL0910G-5, Focus Diagnostics, USA). Sera were also tested for the presence of anti-HSV-2 IgG using HerpeSelect 2 ELISA kits (Cat. No. EL0910G-5, Focus Diagnostics, USA). Both kits are approved for laboratory diagnosis of anti-HSV-1 IgG and anti-HSV-2 IgG by the United States Food and Drug Administration.

For both kits, analysis and results were interpreted according to the manufacturer’s instructions: sera with optical density index values (cut-off) < 0.90 were considered negative, values > 1.10 were considered positive, and values ranging between 0.90 and 1.10 were considered equivocal. All equivocal specimens were retested for final result. Those that remained equivocal were reported as equivocal.

### Oversight

Hamad Medical Corporation and Weill Cornell Medicine-Qatar Institutional Review Boards approved this retrospective study with a waiver of informed consent. The study was reported following the Strengthening the Reporting of Observational Studies in Epidemiology (STROBE) guidelines (Additional file 1: Table S1).

### Statistical analysis

Frequency distributions were used to characterize study participants. Equivocal specimens were excluded from analysis. Probability weights were calculated based on CMW population distribution by age, nationality, and QRCS center per the QRCS registered-user database [20]. The probability weights were applied in the analyses to adjust for participants’ unequal selection.

Associations with antibody positivity for each of HSV-1 and HSV-2 were explored using Chi-square test and univariable logistic regression analyses. Any variable with *p* value ≤ 0.2 in the univariable regression analysis was included in the multivariable model. A *p* value ≤ 0.05 in the multivariable analysis was considered to provide evidence for a statistically significant association. Unadjusted and adjusted odds ratios (ORs and AORs, respectively) along with their respective 95% confidence intervals (CIs) and *p* values were reported. 95% CIs were not adjusted for multiplicity; thus, they should not be used to infer definitive differences between groups. Interactions were not considered. All analyses were conducted using SPSS version 27.0 (Armonk, NY, USA).

## Results

Characteristics of the study population are summarized in Table 1. A total of 2,641 male CMWs consented to participate in the original SARS-CoV-2 survey, but only 2,612 (98.9%) sera specimens were available to be tested for HSV-1 and HSV-2 antibodies and included in the study. The majority of study participants were 30–39 years of age (37.1%) with a median age of 35.0 years (interquartile range 29.0–42.0 years). Of study participants, 42.1% completed intermediate or lower educational attainment, and 43.3% attended high school or vocational training. Of study participants, 27.5% were Indians, 24.0% Bangladeshis, and 21.6% Nepalese, representative of the wider CMW population in Qatar [22]. Each other nationality contributed < 10% of the total sample. Over half of the sample consisted of technical and construction workers such as carpenters, crane operators, electricians, masons, mechanics, painters, plumbers, and welders. About 5% of participants held higher professional positions such as architects, designers, engineers, operation managers, and supervisors.

**Table 1:** Characteristics of study participants and outcome of HSV-1 and HSV-2 serological testing

Characteristics	Tested	HSV-1 antibody positive			HSV-2 antibody positive		
	N (%)	N	%^a^ (95% CI)^a^	Chi-square *p* value	N	%^a^ (95% CI)^a^	Chi-square *p* value
*Age (years)*							
≤ 29	746 (28.6)	598	81.8 (78.8–84.6)	0.228	65	9.0 (7.0–11.3)	0.017
30–39	968 (37.1)	808	84.3 (82.0–86.4)		107	11.2 (9.4–13.3)	
40–49	547 (20.9)	467	86.5 (83.4–89.3)		73	12.9 (10.2–16.0)	
50–59	260 (10.0)	219	85.0 (79.1–89.7)		38	14.1 (9.5–19.9)	
60+	91 (3.5)	79	87.0 (73.7–95.1)		17	22.2 (11.2–37.1)	
*Nationality*							
All other nationalities^b^	231 (8.8)	217	95.4 (91.5–97.9)	< 0.001	52	23.2 (17.5–29.9)	< 0.001
Bangladeshi	626 (24.0)	568	91.4 (89.1–93.4)		73	11.2 (8.9–13.9)	
Egyptian	90 (3.4)	85	96.3 (89.7–99.2)		7	7.2 (2.7–15.1)	
Filipino	102 (3.9)	78	75.7 (64.0–85.2)		9	8.6 (3.2–17.7)	
Indian	717 (27.5)	510	72.6 (69.2–75.7)		87	12.7 (10.4–15.2)	
Nepalese	565 (21.6)	505	90.2 (87.5–92.5)		44	7.6 (5.5–10.1)	
Pakistani	136 (5.2)	125	92.1 (86.0–96.2)		12	8.8 (4.5–15.2)	
Sri Lankan	145 (5.6)	83	58.8 (49.4–67.8)		16	10.1 (5.3–17.0)	
*QRCS center (catchment area within Qatar)*							
Fereej Abdel Aziz (Doha-East)	612 (23.4)	496	82.0 (78.7–85.0)	0.354	58	9.6 (7.4–12.2)	0.358
Hemaila (South-West; “Industrial Area”)	966 (37.0)	813	84.9 (82.6–97.0)		113	11.8 (10.0–13.9)	
Mesaimeer (Doha-South)	800 (30.6)	672	85.0 (82.4–87.4)		93	11.7 (9.6–14.1)	
Zekreet (North-West)	234 (9.0)	190	82.8 (70.6–91.4)		36	15.5 (7.4–27.4)	
*Educational attainment*							
Primary or lower	628 (25.0)	561	89.5 (86.8–91.8)	< 0.001	88	13.5 (10.9–16.5)	0.128
Intermediate	429 (17.1)	377	90.0 (86.8–92.7)		48	10.2 (7.5–13.4)	
Secondary/High school/Vocational	1088 (43.3)	880	82.5 (80.1–84.7)		118	11.5 (9.6–13.5)	
University	369 (14.7)	265	71.3 (66.0–76.1)		35	8.8 (6.0–12.3)	
*Occupation*							
Professional workers^c^	136 (5.3)	92	67.2 (58.1–75.4)	< 0.001	11	8.1 (3.9–14.3)	0.113
Food & beverage workers	91 (3.6)	71	80.0 (69.6–88.1)		10	11.3 (5.3–20.3)	
Administration workers	82 (3.2)	61	71.8 (60.5–81.4)		2	2.6 (0.3–9.2)	
Retail workers	171 (6.7)	142	87.2 (81.1–91.9)		19	11.3 (7.0–17.1)	
Transport workers	430 (16.8)	348	80.9 (76.8–84.6)		58	12.3 (9.3–15.9)	
Cleaning workers	104 (4.1)	91	90.0 (82.4–95.1)		11	8.2 (3.6–15.5)	
Technical and construction workers^d^	1314 (51.3)	1123	86.5 (84.5–88.2)		151	11.6 (10.0–13.5)	
Security workers	60 (2.3)	56	94.9 (85.9–98.9)		6	10.2 (3.8–20.8)	
Other workers^e^	175 (6.8)	148	86.2 (80.2–91.0)		23	13.4 (8.7–19.4)	
Total (%, 95% CI)	2612 (100.0)	2171	84.2 (82.8–85.6)	NA	300	11.4 (10.1–12.6)	NA

CI, confidence interval; HSV-1, herpes simplex virus type 1; HSV-2, herpes simplex virus type 2; NA, not applicable; QRCS, Qatar Red Crescent Society

^a^Percentage of positive out of those tested weighted by age, nationality, and QRCS center. Equivocal specimens were excluded from the analysis

^b^Includes all other nationalities of craft and manual workers residing in Qatar

^c^Includes architects, designers, engineers, operation managers, and supervisors among other professions

^d^Includes carpenters, construction workers, crane operators, electricians, foremen, maintenance/air conditioning/cable technicians, masons, mechanics, painters, pipe-fitters, plumbers, and welders among other professions

^e^Includes barbers, firefighters, gardeners, farmers, fishermen, and physical fitness trainers among other professions

### Herpes simplex virus type 1

**Fig. 1:**
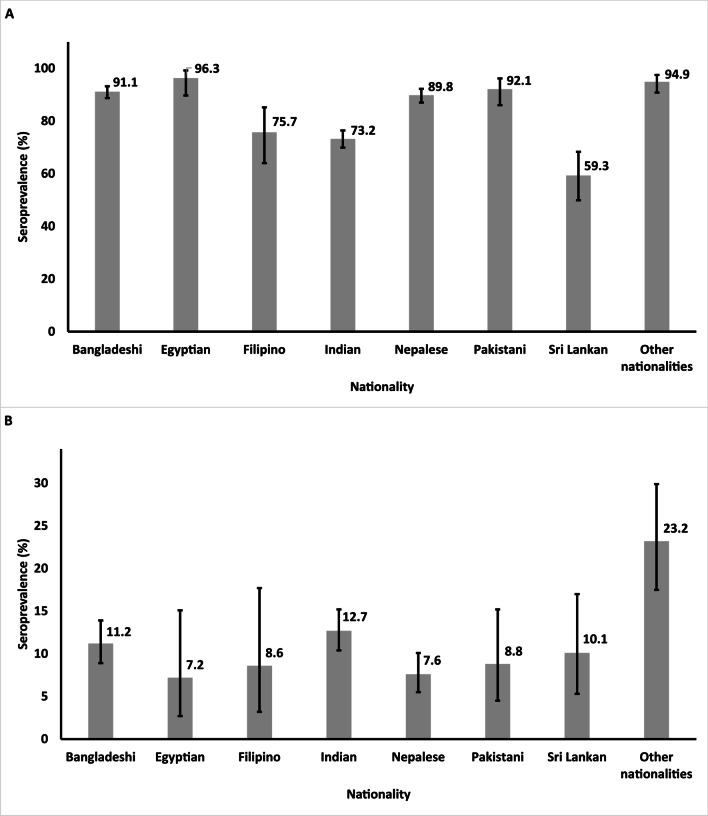
HSV-1 (**A**) and HSV-2 (**B**) seroprevalence by nationality among the craft and manual worker population in Qatar. HSV-1, herpes simplex virus type 1; HSV-2, herpes simplex virus type 2

In total, 2,612 serum specimens were tested for HSV-1 antibodies (Table 1). The serological testing identified 2,171 sera as positive, 403 as negative, and 38 as equivocal. HSV-1 seroprevalence among the CMW population was estimated at 84.2% (95% CI 82.8–85.6%), after excluding equivocal specimens from both numerator and denominator of the ratio defining seroprevalence. Seroprevalence was highest among Egyptians at 96.3% (95% CI 89.7–99.2%) and lowest among Sri Lankans at 58.8% (95% CI 49.4–67.8%) (Fig. 1A).

HSV-1 seropositivity was independently associated with each of nationality, educational attainment, and occupation (Table 2). Compared to “other” nationalities, significant differences in seropositivity were observed among Bangladeshis (AOR 0.36; 95% CI 0.17–0.77), Filipinos (AOR 0.20; 95% CI 0.08–0.49), Nepalese (AOR 0.31; 95% CI 0.14–0.66), Indians (AOR 0.11; 95% CI 0.05–0.23), Pakistanis (AOR 0.37; 95% CI 0.14–0.96), and Sri Lankans (AOR 0.06; 95% CI 0.03–0.12). Compared to workers with primary or lower education, AOR was 0.68 (95% CI 0.49–0.94) for workers with secondary/high school/vocational level education and 0.34 (95% CI 0.22–0.52) for workers with university level education. Compared to professional workers, AOR was 2.03 (95% CI 1.06–3.90) for retail workers. No association was found for age and QRCS center (proxy of catchment area/geographic location).

**Table 2:** Associations with HSV-1 and HSV-2 seropositivity

Characteristics	Anti-HSV-1 positive					Anti-HSV-2 positive				
	Univariable regression analysis			Multivariable regression analysis		Univariable regression analysis			Multivariable regression analysis	
	OR^a^ (95% CI^a^)	*p* value	F test *p* value^b^	AOR^a^ (95% CI^a^)	*p* value^c^	OR^a^ (95% CI^a^)	*p* value	F test *p* value^b^	AOR^a^ (95% CI^a^)	*p* value^c^
*Age (years)*										
≤ 29	1.00		0.218	–	–	1.00		0.027	1.00	
30–39	1.19 (0.92–1.53)	0.210		–	–	1.29 (0.94–1.78)	0.120		1.36 (0.97–1.89)	0.076
40–49	1.43 (0.99–1.95)	0.053		–	–	1.52 (1.06–2.17)	0.023		1.55 (1.06–2.26)	0.020
50–59	1.25 (0.80–1.96)	0.317		–	–	1.71 (1.06–2.76)	0.029		1.77 (1.07–2.94)	0.028
60+	1.59 (0.64–3.96)	0.363		–	–	2.87 (1.35–6.11)	0.006		2.79 (1.25–6.24)	0.012
*Nationality*										
All other nationalities^d^	1.00		< 0.001	1.00		1.00		< 0.001	1.00	
Bangladeshi	0.51 (0.25–1.05)	0.066		0.36 (0.17–0.77)	0.009	0.41 (0.27–0.62)	< 0.001		0.35 (0.22–0.54)	< 0.001
Egyptian	1.36 (0.35–5.39)	0.659		1.66 (0.41–6.74)	0.475	0.25 (0.10–0.61)	0.002		0.21 (0.08–0.57)	0.002
Filipino	0.15 (0.06–0.35)	< 0.001		0.20 (0.08–0.49)	< 0.001	0.29 (0.12–0.73)	0.009		0.33 (0.13–0.84)	0.021
Indian	0.13 (0.06–0.25)	< 0.001		0.11 (0.05–0.23)	< 0.001	0.47 (0.32–0.70)	< 0.001		0.41 (0.27–0.62)	< 0.001
Nepalese	0.44 (0.21–0.91)	0.026		0.31 (0.14–0.66)	0.003	0.26 (0.17–0.42)	< 0.001		0.23 (0.14–0.37)	< 0.001
Pakistani	0.54 (0.21–1.36))	0.188		0.37 (0.14–0.96)	0.041	0.32 (0.16–0.64)	0.001		0.26 (0.13–0.55)	0.002
Sri Lankan	0.07 (0.03–0.15)	< 0.001		0.06 (0.03–0.12)	< 0.001	0.37 (0.18–0.72)	0.004		0.32 (0.16–0.64)	< 0.001
*QRCS center (catchment area within Qatar)*										
Fereej Abdel Aziz (Doha-East)	1.00		0.377	–	–	1.00		0.239	–	–
Hemaila (South-West; “Industrial Area”)	1.24 (0.95–1.61)	0.118		–	–	1.27 (0.91–1.76)	0.160		–	–
Mesaimeer (Doha-South)	1.05 (0.52–2.14)	0.892		–	–	1.26 (0.89–1.78)	0.185		–	–
Zekreet (North-West)	1.24 (0.94–1.65)	0.113		–	–	1.76 (0.83–3.74)	0.142		–	–
*Educational attainment*										
Primary or lower	1.00		< 0.001	1.00		1.00		0.054	1.00	
Intermediate	0.98 (0.66–1.46)	0.906		1.09 (0.71–1.65)	701	0.72 (0.49–1.06)	0.100		0.74 (0.49–1.09)	0.833
Secondary/High school/Vocational	0.54 (0.40–0.73)	< 0.001		0.68 (0.49–0.94)	0.018	0.83 (0.62–1.12)	0.219		0.77 (0.57–0.92)	0.012
University	0.30 (0.21–0.43)	< 0.001		0.34 (0.22–0.52)	< 0.001	0.61 (0.39–0.96)	0.032		0.43 (0.28–0.76)	< 0.001
*Occupation*										
Professional workers^e^	1.00		< 0.001	1.00		1.00		0.251	–	–
Food & beverage workers	2.02 (0.98–3.93)	0.056		0.89 (0.42–1.90)	0.770	1.43 (0.55–3.73)	0.469		–	–
Administration workers	1.27 (0.68–2.37)	0.452		0.83 (0.42–1.66)	0.602	0.29 (0.06–1.44)	0.129		–	–
Retail workers	3.33 (1.84–6.02)	< 0.001		2.03 (1.06–3.90)	0.034	1.47 (0.65–3.33)	0.353		–	–
Transport workers	2.10 (1.34–3.30)	0.001		1.21 (0.71–3.70)	0.483	1.63 (0.80–3.34)	0.182		–	–
Cleaning workers	4.28 (2.03–9.01)	< 0.001		1.61 (0.70–3.67)	0.261	1.05 (0.40–2.78)	0.920		–	–
Technical and construction workers^f^	3.15 (3.09–4.73)	< 0.001		1.47 (0.90–2.40)	0.123	1.54 (0.78–3.02)	0.212		–	–
Security workers	8.05 (2.56–25.43)	< 0.001		2.05 (0.61–6.91)	0.245	1.40 (0.49–4.00)	0.534		–	–
Other workers^g^	3.15 (1.77–5.59)	< 0.001		1.45 (0.76–2.79)	0.259	1.83 (0.84–4.01)	0.131		–	–

AOR, adjusted odds ratio; CI, confidence interval; HSV-1, herpes simplex virus type 1; HSV-2, herpes simplex virus type 2; OR, odds ratio; QRCS, Qatar Red Crescent Society

^a^Estimates weighted by age, nationality, and QRCS center

^b^Covariates with *p* value ≤ 0.2 in the univariable analysis were included in the multivariable analysis

^c^Covariates with *p* value ≤ 0.05 in the multivariable analysis were considered to provide statistically significant evidence for an association with antibody positivity

^d^Includes all other nationalities of craft and manual workers residing in Qatar

^e^Includes architects, designers, engineers, operation managers, and supervisors among other professions

^f^Includes carpenters, construction workers, crane operators, electricians, foremen, maintenance/air conditioning/cable technicians, masons, mechanics, painters, pipe-fitters, plumbers, and welders among other professions

^g^Includes barbers, firefighters, gardeners, farmers, fishermen, and physical fitness trainers among other professions

### Herpes simplex virus type 2

In total, 2,612 serum specimens were tested for HSV-2 antibodies (Table 1). The serological testing identified 300 sera as positive, 2,250 as negative, and 62 as equivocal. HSV-2 seroprevalence among the CMW population was estimated at 11.4% (95% CI 10.1–12.6%), after excluding equivocal specimens from both numerator and denominator of the ratio defining seroprevalence. Seroprevalence was highest among “other” nationalities at 23.2% (95% CI 17.5–29.9%) and lowest among Egyptians at 7.2% (95% CI 2.7–15.1%) (Fig. 1B).

HSV-2 seropositivity was independently associated with each of age, nationality, and educational attainment (Table 2). The AOR increased with age. Compared to those aged ≤ 29 years, AOR was 1.55 (95% CI 1.06–2.26) for those aged 40–49 years and 2.79 (95% CI 1.25–6.24) for those aged over 60 years. Compared to “other” nationalities, significant differences in seropositivity were observed among all nationalities. Compared to workers with primary or lower education, AOR was 0.77 (95% CI 0.57–0.92) for workers with secondary/high school/vocational level education and 0.43 (95% CI 0.28–0.76) for workers with university level education. No association was found for QRCS center and occupation.

## Discussion

HSV-1 seroprevalence was overall high in the CMW population of Qatar at a level that exceeded 80%. However, there was substantial variation in seroprevalence by nationality ranging from 58.8% among Sri Lankans to 96.3% among Egyptians. Seroprevalence in each nationality group was similar to the seroprevalence observed in the country of origin [25–27], but not to that of Qataris [28]. This may be explained by these workers arriving to Qatar only within the last decade and not being permanent residents of Qatar. Since HSV-1 is mostly acquired orally during childhood [1, 25, 26, 29–33], these workers have likely acquired the infection before coming to Qatar. Seroprevalence did not vary by age, further supporting that HSV-1 acquisition occurred in childhood. Against paucity of HSV-1 seroprevalence data in South Asia [25], this study provides HSV-1 seroprevalence data for different South Asian nationalities, including what appears to be the first one for Nepalese in the literature.

Remarkably, this study confirms an unexplained anomaly in global HSV-1 seroprevalence data [27, 28], the low HSV-1 seroprevalence in Sri Lanka and India compared to what is expected for countries of similar socio-economic status [25, 26, 28–30]. HSV-1 seroprevalence among Sri Lankans and Indians was lowest of all nationalities, supporting the relatively low HSV-1 seroprevalence reported previously in this part of the world [25, 34, 35].

HSV-2 seroprevalence varied by nationality but was overall at about 10%, in the intermediate range compared to global data on HSV-2 seroprevalence among men [1, 36–42]. HSV-2 seroprevalence increased with age, consistent with continuing acquisition of this infection in adulthood. These findings suggest presence of genital herpes and genital ulcer disease burden in the CMW population, but this burden remains untackled and poorly documented in context of limited programs for sexual health and sexually transmitted infections.

HSV-2 seroprevalence in each nationality was similar to the seroprevalence in the country of origin, and higher than that observed among Qataris and in other countries of the Middle East and North Africa [41, 43, 44]. This is also explained by these workers arriving only within the last decade to Qatar. Against paucity of HSV-2 seroprevalence data in South Asia [36], this study provides HSV-2 seroprevalence data for different South Asian nationalities, including what appears to be the first one for Nepalese in the literature.

HSV-1 seroprevalence as well as HSV-2 seroprevalence were lower with higher socio-economic status as measured by education and occupation. This finding supports the inverse association observed in different countries between HSV-1 and HSV-2 infections and socio-economic status [28, 45, 46].

This study has limitations. While the study design was intended to be based on probability-based sampling of the total CMW population in Qatar, operational challenges forced instead a systematic sampling of QRCS attendees supplemented with probability-based weights to generate an estimate that is representative of the wider CMW population. To ensure representation of small age-nationality strata, all attendees in these strata (not only every 4^th^ attendee) were approached to participate towards the end of the study.

Operational challenges made it also difficult to track and maintain consistent logs of the response rate by the nurses in these QRCS centers. Therefore, an exact estimate of the response rate could not be ascertained, though it was estimated based on the interviewers’ experience at > 90%. While it is possible that the recruitment scheme may have affected the generalizability of study findings, this is less likely considering that CMWs attend these centers at a high volume that exceeds 5,000 patients per day and for a range of services beyond illness such as periodic health certifications, vaccinations, and pre-travel SARS-CoV-2 testing.

Only socio-demographic variables were collected, not including sexual behavior data. Collecting sexual behavior data is difficult in this culturally conservative setting, but this difficulty highlights the value of using HSV-2 seroprevalence as a proxy biomarker of population sexual risk behavior, as demonstrated previously [44, 47]. We used a unified statistical analysis plan for both infections that employed logistic regression analyses with the associations estimated in terms of ORs. However, for highly prevalent infections such as HSV-1, the ORs are too sensitive to even small changes in seroprevalence. Too large or too small ORs may not necessarily imply large differences in actual seroprevalence.

Since the sample consisted exclusively of men, it was not possible to investigate differences in seroprevalence by sex. However, existing evidence from systematic reviews and meta-regressions suggests that HSV-2 seroprevalence is approximately 30–40% lower among men compared to women [36–41]. On the other hand, HSV-1 seroprevalence does not typically show differences by sex, as it is primarily acquired orally during childhood [25, 26, 29–33].

Though we used quality, validated, and widely used FDA-approved commercial assays, existing data suggests potential population variation in assay sensitivity and specificity [48], which may affect the estimated seroprevalence. A number of specimens tested equivocal even after repeat testing and were excluded from analysis. However, the number of equivocal specimens was relatively small to appreciably affect study findings.

## Conclusions

In conclusion, over 80% of CMWs in Qatar are infected with HSV-1, and over 10% are infected with HSV-2. These findings highlight the need for monitoring of trends in HSV-1 and HSV-2 seroprevalence and etiological surveillance of genital ulcer disease, genital herpes, and other HSV-related morbidities. The findings stress the need for programs to tackle sexually transmitted infections and address broader sexual health needs of this population. The findings also support the relevance of HSV prophylactic and therapeutic vaccine development [49], as well as further epidemiological research on HSV infection in this population to address its healthcare needs.

### Supplementary Information

**Additional file 1: Table S1**. STROBE checklist for cross-sectional studies.

## Data Availability

All data are available in aggregate form within the manuscript.
